# Pyrosequencing Analysis Reveals Changes in Intestinal Microbiota of Healthy Adults Who Received a Daily Dose of Immunomodulatory Probiotic Strains

**DOI:** 10.3390/nu7063999

**Published:** 2015-05-26

**Authors:** Julio Plaza-Díaz, Jose Ángel Fernández-Caballero, Natalia Chueca, Federico García, Carolina Gómez-Llorente, María José Sáez-Lara, Luis Fontana, Ángel Gil

**Affiliations:** 1 Department of Biochemistry and Molecular Biology II, School of Pharmacy, University of Granada, Granada 18071, Spain; E-Mails: jrplaza@ugr.es (J.P.-D.); gomezll@ugr.es (C.G.-L.); fontana@ugr.es (L.F.); 2 Institute of Nutrition and Food Technology “José Mataix”, Biomedical Research Center, University of Granada, Armilla 18100, Spain; 3 Department of Microbiology, Complejo Hospitalario Universitario de Granada, Instituto de Investigación Biosanitaria (IBS), Granada 18012, Spain; E-Mails: jafr7@correo.ugr.es (J.A.F.-C.); nchueca@ugr.es (N.C.); fegarcia@ugr.es (F.G.); 4 Department of Biochemistry and Molecular Biology I, School of Sciences, University of Granada, Granada 18071, Spain; E-Mail: mjsaez@ugr.es

**Keywords:** gut, healthy adults, high-throughput nucleotide sequencing, microbiota, probiotics

## Abstract

The colon microbiota plays a crucial role in human gastrointestinal health. Current attempts to manipulate the colon microbiota composition are aimed at finding remedies for various diseases. We have recently described the immunomodulatory effects of three probiotic strains (*Lactobacillus rhamnosus* CNCM I-4036, *Lactobacillus paracasei* CNCM I-4034, and *Bifidobacterium breve* CNCM I-4035). The goal of the present study was to analyze the compositions of the fecal microbiota of healthy adults who received one of these strains using high-throughput 16S ribosomal RNA gene sequencing. *Bacteroides* was the most abundant genus in the groups that received *L. rhamnosus* CNCM I-4036 or *L. paracasei* CNCM I-4034. The Shannon indices were significantly increased in these two groups. Our results also revealed a significant increase in the *Lactobacillus* genus after the intervention with *L. rhamnosus* CNCM I-4036. The initially different colon microbiota became homogeneous in the subjects who received *L. rhamnosus* CNCM I-4036. While some orders that were initially present disappeared after the administration of *L. rhamnosus* CNCM I-4036, other orders, such as *Sphingobacteriales*, *Nitrospirales*, *Desulfobacterales*, *Thiotrichales*, and *Synergistetes*, were detected after the intervention. In summary, our results show that the intake of these three bacterial strains induced changes in the colon microbiota.

## 1. Introduction

The human colon microbiota is a complex ecosystem that is composed of approximately 10^14^ bacterial cells [1], which is ten times the number of cells in the human body, and it has been suggested to encode 100-fold more unique genes than the human genome [2]. Approximately 400–500 bacterial species comprise the colon microbiota [3]. The colon microbiota has a profound influence on human physiology and nutrition and plays a crucial role in human gastrointestinal (GI) health, affecting metabolism and the immune system and protecting against pathogens while modulating GI development [4,5].

Current attempts to manipulate the GI tract microbiota are focused on finding remedies for several health disorders, including infections and inflammatory, allergic, and immunologic conditions. Probiotics are consumed in different forms, such as yogurt, cheese and fermented foods, as a regular part of the human diet and as treatments for different GI tract dysfunctions [6]. However, the actual ability of probiotics to affect gut microorganisms is still under debate, because although it has been confirmed in several studies, [7] numerous confounding elements exist, such as diverse consumer’s susceptibilities to probiotic intake and marked differences in probiotic products (e.g., dissimilarities in microbial strains, concentrations of viable cells, and product formulations) [8].

The development of high-throughput 16S ribosomal RNA gene sequencing techniques has accelerated the knowledge of gut microbiome diversity [9]. Pyrosequencing allows for the determination of the entire phylogenetic spectrum, taxonomic characterization, and the flexibility to analyze populations at different taxonomic levels. Thus, 16S rRNA gene-based barcoded pyrosequencing has been extensively used for the characterization of gut microbial communities in healthy and diseased individuals [10,11,12] and has also been used to study the effects of dietary intervention on the colon microbiota [10,13,14].

Recently, we described a multicenter, randomized, double-blind, placebo-controlled trial performed to assess the effects of three novel probiotic strains (*Lactobacillus rhamnosus* CNCM I-4036, *Lactobacillus paracasei* CNCM I-4034, and *Bifidobacterium breve* CNCM I-4035) on healthy volunteers, including evaluations of tolerance, safety, persistence in the gut, and immunomodulatory effects [15]. We found intestinal colonization in the volunteers who received *L. rhamnosus* CNCM I-4036 and that the administration of *B. breve* CNCM I-4035 results in a significant increase in the secretory IgA level. Further, we demonstrated that IL-4 and IL-10 levels increase whereas IL-12 decreases in the sera of volunteers treated with any of the three strains. The consumption of these three bacterial strains is safe and leads to varying degrees of immunomodulatory effects [15].

The aim of the present study was to analyze the composition of the human fecal microbiota of healthy adults who received daily doses of either a placebo or one of the three aforementioned probiotic strains using high-throughput 16S ribosomal RNA gene sequencing. Additionally, we investigated the impact of probiotic administration on intestinal colonization, which might contribute to the current understanding of the immunomodulatory effects of these three strains [15].

## 2. Materials and Methods

### 2.1. Ethical Statement

The probiotic strains *Lactobacillus paracasei* CNCM I-4034, *Bifidobacterium breve* CNCM I-4035 and *Lactobacillus rhamnosus* CNCM I-4036 were obtained from the feces of breast-fed newborns, as previously described (SETOPROB study) [15,16,17]. These strains were assayed for enzymatic activity and carbohydrate utilization, and they were deposited in the Collection Nationale de Cultures de Microorganismes (CNCM) of the Institute Pasteur [18]. This study followed the guidelines of the Declaration of Helsinki, and all procedures involving human subjects were approved by the ethics committees of the University of Granada, Murcia and Valencia. Written informed consent was obtained from the subjects after a careful explanation of the nature of the study. Additionally, this trial was registered at www.clinicaltrials.gov as NCT01479543.

### 2.2. Subjects and Experimental Design

Twenty-five healthy volunteers underwent a 15-day washout period (*t*_1_), after which they were randomly and blindly divided into five groups and received either a placebo, a capsule containing 9 × 10^9^ CFUs of one of the three strains, or a capsule containing 9 × 10^9^ CFUs of a mixture of *Bifidobacterium breve* CNCM I-4035 and *Lactobacillus rhamnosus* CNCM I-4036 each day for 30 days (*t*_2_). The intervention period was followed by a second washout of another 15 days (*t*_3_) for the five different groups. The patients did not consume any fermented milk products, or products containing either probiotic bacteria or prebiotics for the entire duration of the study [15]. There were no additional dietary restrictions.

### 2.3. Fecal Samples, DNA Extraction and 454 Pyrosequencing

Fecal samples were collected from each volunteer at *t*_1_, *t*_2_, and *t*_3_ under anaerobic conditions by using the Anaerogen Compact System (Oxoid, Basingstoke, UK). The system consists of a plastic pouch and a paper gas generating sachet. The paper sachet contained ascorbic acid and activated carbon, which reacts with air. Oxygen is rapidly absorbed and carbon dioxide is produced. The fecal samples were placed inside the plastic pouch together with the sachet, and the pouch was immediately sealed. Samples were kept under anaerobic conditions for 4 h, and then transferred to −80°C until analysis.

Fecal samples were homogenized in a Stomacher-400 blender. DNA was extracted using a QIAamp DNA Stool Mini Kit (QIAGEN, Barcelona, Spain) as directed by the manufacturer, with the exception that samples were mixed with the lysis buffer and incubated at a temperature of 95°C instead of 70°C to ensure lysis of both Gram-positive and negative bacteria. Quantification was conducted with a NanoDrop ND-1000 spectrophotometer (Thermo Fisher Scientific, DE, USA). Extracted DNA samples from each participant for each collection period were sent to the Department of Microbiology, University Hospital San Cecilio (Granada, Spain). The amplification of a 600-bp sequence in the variable region V1-V3 of the 16S rRNA gene was performed using barcoded primers. PCR was performed in a total volume of 15 µL for each sample containing the universal 27F and Bif16S-F forward primers (10 μmol/L) at a 9:1 ratio, respectively, and the barcoded universal reverse primer 534R (10 µmol/L) in addition to dNTP mix (10 mmol/L), FastStart 10× buffer with 18 mmol/L of MgCl_2_, FastStart HiFi polymerase (5 U in 1 mL), and 2 µL of genomic DNA. The dNTP mix, FastStart 10× buffer with MgCl_2_, and FastStart HiFi polymerase were included in a FastStart High Fidelity PCR System, dNTP Pack (Roche Applied Science). The PCR conditions were as follows: 95 °C for 2 min, 30 cycles of 95 °C for 20 s, 56 °C for 30 s, and 72 °C for 5 min, and final step at 4 °C. After PCR, amplicons were further purified using AMPure XP beads (Beckman-Coulter) to remove smaller fragments. DNA concentration and quality were measured using a Quant-iT™ PicoGreen^®^ dsDNA Assay Kit. Finally, the PCR amplicons were combined in equimolar ratios to create a DNA pool (10^9^ DNA molecules) that was used for clonal amplification (emPCR) and pyrosequencing according to the manufacturer’s instructions.

Pyrosequencing of the PCR amplicons was performed using a Roche/454 GS Titanium technology platform (Branford, CT, USA). After the sequencing was completed, all reads were scored for quality, and any poor quality and short reads were removed.

### 2.4. Taxonomic Analysis

Sequences were selected to estimate the total bacterial diversity of the DNA samples in a comparable manner and were trimmed to remove barcodes, primers, chimeras, plasmids, mitochondrial DNA and any non-16S bacterial reads and sequences <150 bp. MG-RAST (metagenomics analysis server) [19] with the Ribosomal Database Project (RDP) were used for analyses of all sequences. The input processing steps in MG-RAST included demultiplexing, quality filtering, length filtering, dereplication, and removal of model organism sequences. The preprocessing options and details of data provided were: FASTQ sequences were filtered using a dynamic trimming. Fifteen was the specific lowest Phred quality score that was counted as a high-quality base and the sequences containing 5 bases below the value score 15 were trimmed.

The raw microbiologic data were reported as relative abundances at the taxonomic levels of phylum, family, and genus. The Shannon index, which is based on species richness (the number of species present) and species abundance (the number of individuals per species), was calculated using all three times *t*_1_, *t*_2_ and *t*_3_. Differences among times were compared by using the Mann–Whitney *U*-test.

### 2.5. Statistical Analysis

Because the data of relative abundance were not normally distributed, median and ranges were used to express those results. The differences in the relative abundances of the families and genera for each sample were computed using the Mann–Whitney *U*-test. The clustering of the colon microbiota within the *L. rhamnosus* CNCM I-4036 group was calculated using a principal component analysis (PCoA). In the PCoA plot the data were analyzed with RDP. The data were normalized to values between 0 and 1 and Euclidean distance was measured. A PERMANOVA analysis was done using the PRIMER 7 software. Finally, a tree diagram was created with the MG-RAST software, which allowed for comparisons of the datasets at the level/rank of order.

## 3. Results

### 3.1. Subject Information and Sequencing Coverage

We randomly selected 25 healthy adult volunteers and placed five into each group; however, two individuals were excluded due to insufficient fecal samples. Accordingly, the number of subjects was decreased to twenty-three (14 males and 9 females) after the DNA extractions and pyrosequencing analysis. We evaluated five healthy volunteers in the placebo, *Lactobacillus rhamnosus* CNCM I-4036, and *Lactobacillus paracasei* CNCM I-4034 groups and four healthy volunteers in the mixture and *Bifidobacterium breve* CNCM I-4035 groups. A total of 343,063 sequences were generated using 454 pyrosequencing, and the number of sequences per sample varied from 1725 to 10,569 (median of 4782) (Table 1).

**Table 1:** Volunteer information.

Subject code	Capsule	Age	Gender	Number of sequences		
				*t*_1_	*t*_2_	*t*_3_
A	Placebo	20	Female	3772	4924	3170
B	Placebo	27	Female	4581	5864	4674
C	Placebo	30	Male	6108	4819	5564
D	Placebo	29	Male	4005	5455	5214
E	Placebo	27	Male	7231	6276	4801
F	*L. rhamnosus* CNCM I-4036	28	Female	1725	7814	6949
G	*L. rhamnosus* CNCM I-4036	20	Female	4971	4506	4877
H	*L. rhamnosus* CNCM I-4036	21	Male	4052	3795	4146
I	*L. rhamnosus* CNCM I-4036	29	Female	3538	3595	4564
J	*L. rhamnosus* CNCM I-4036	20	Male	3523	5645	5221
K	*B. breve* CNCM I-4035	21	Male	10,569	4525	4782
L	*B. breve* CNCM I-4035	20	Male	4924	4976	4381
M	*B. breve* CNCM I-4035	26	Male	3888	1940	4772
N	*B. breve* CNCM I-4035	35	Male	5708	5627	5370
O	*B. breve* CNCM I-4035 and *L. rhamnosus* CNCM I-4036	20	Female	7098	8277	6383
P	*B. breve* CNCM I-4035 and *L. rhamnosus* CNCM I-4036	30	Male	7860	3646	5009
Q	*B. breve* CNCM I-4035 and *L. rhamnosus* CNCM I-4036	25	Male	3437	4506	4486
R	*B. breve* CNCM I-4035 and *L. rhamnosus* CNCM I-4036	27	Female	5707	3011	3397
S	*L. paracasei* CNCM I-4034	28	Male	6095	4015	4651
T	*L. paracasei* CNCM I-4034	26	Male	9256	5426	2931
U	*L. paracasei* CNCM I-4034	25	Female	3926	5271	6547
V	*L. paracasei* CNCM I-4034	26	Male	3726	4208	3523
W	*L. paracasei* CNCM I-4034	31	Female	2753	8642	2435

Volunteers received either a placebo, a capsule containing 9 × 10^9^ CFUs of one of the three strains, or a capsule containing 9 × 10^9^ CFUs of a mixture of *Bifidobacterium breve* CNCM I-4035 and *Lactobacillus rhamnosus* CNCM I-4036 each day for 30 days. *t*_1_, first 15-day washout period; *t*_2_, intervention period; and *t*_3_, second washout for another 15 days.

**Figure 1:**
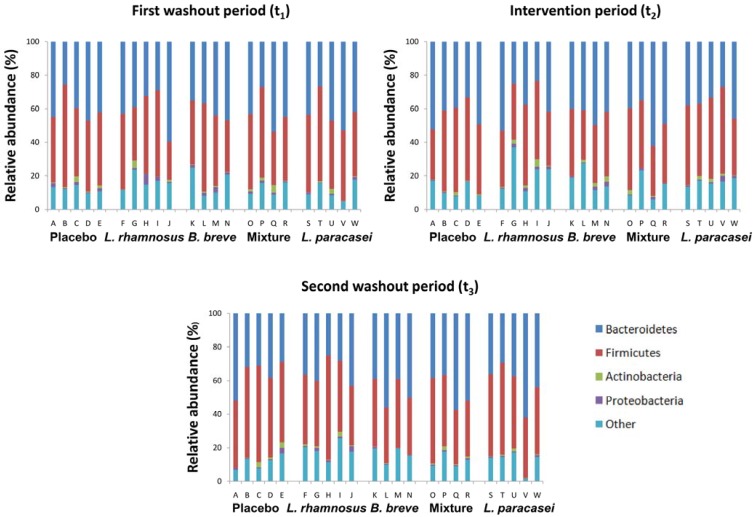
Compositions of fecal microbiota in 23 healthy adults at the phylum level. The subjects were divided into five groups and received either a placebo, a capsule containing 9 × 10^9^ CFUs of one of the three strains, or a capsule containing 9 × 10^9^ CFUs of a mixture of *B. breve* CNCM I-4035 and *L. rhamnosus* CNCM I-4036 each day for 30 days. Each column represents 1 healthy adult, as described in Table 1.

Our analysis of the intestinal microbiota revealed that the volunteers enrolled in this study possessed colon microbiota compositions at *t*_1_ which did not associate with any intestinal disorder. At the phylum level (Figure 1), the prevalences of the four major phyla (*Firmicutes*, *Bacteroidetes*, *Proteobacteria* and *Actinobacteria*) varied considerably among the samples collected at different time points. All four phyla were present, varying from less than 2% to more than 50% in abundance.

At the genus level (Table 2), Bacteroides was the most abundant genus in all of the study groups, and the levels of *Faecalibacterium*, *Alistipes*, *Prevotella*, *Clostridium*, *Eubacterium*, *Ruminococcus*, *Phascolarctobacterium*, *Parabacteroides*, *Veillonella*, *Butyrivibrio*, *Akkermansia*, *Bifidobacterium*, *Lactobacillus*, and *Bacillus* were found to vary at the different time points of the study, as extensively described elsewhere [20].

**Table 2:** Phylogenetic analysis at genus level.

Capsule		Genus			
			First washout	Intervention	Second washout
Placebo		*Bacteroides*	18.3 (7.5–37.9)	15.9 (10.8–27.1)	12.5 (6.4–29.9)
		*Faecalibacterium*	10.5 (7.3–15.5)	11.0 (6.4–19.8)	15.1 (7.9–19.8)
		*Alistipes*	7.0 (4.6–11.2)	5.2 (3.4–13.8)	5.7 (3.1–14.3)
		*Prevotella*	7.1 (0.0–13.2)	16.6 (0.0–25.8)	2.4 (0.0–13.3)
		*Clostridium*	6.9 (1.9–8.7)	4.9 (2.6–10.6)	6.1 (3.6–9.3)
		*Eubacterium*	4.0 (1.6–7.1)	5.1 (1.9–7.3)	3.5 (1.6–5.1)
		*Ruminococcus*	1.8 (0.8–8.3)	1.8 (1.2–4.1)	4.6 (2.1–12.1)
		*Phascolarctobacterium*	1.9 (0.0–7.3)	1.8 (0.0–3.6)	1.8 (0.0–4.7)
		*Parabacteroides*	1.9 (1.2–4.5)	1.6 (1.0–5.8)	2.8 (2.4–3.6)
		*Veillonella*	0.8 (0.2–5.2)	0.4 (0.0–4.8)	1.4 (0.4–2.2)
		*Butyrivibrio*	1.1 (0.9–2.6)	1.3 (0.7–2.0)	1.6 (0.2–3.1)
		*Akkermansia*	0.6 (0.0–2.9)	0.0 (0.0–0.1)	0.0 (0.0–1.8)
		*Bifidobacterium*	0.4 (0.1–0.6)	0.2 (0.0–0.8)	0.0 (0.0–0.8)
		*Lactobacillus*	0.0 (0.0–0.5)	0.0 (0.0–0.4)	0.0 (0.0–0.8)
		*Bacillus*	0.0 (0.0–0.2)	0.2 (0.0–0.4)	0.1 (0.0–0.6)
*Lactobacillus rhamnosus* CNCM I-4036		*Bacteroides*	18.9 (8.8–32.0)	13.0 (5.4–29.1)	14.8 (5.4–23.6)
		*Faecalibacterium*	9.9 (3.6–22.4)	6.3 (2.8–20.8)	8.3 (4.5–16.8)
		*Alistipes*	5.5 (0.1–7.0)	4.0 (2.9–15.1)	4.7 (2.1–9.7)
		*Prevotella*	7.1 (0.0–38.1)	4.2 (0.1–26.5)	5.1 (0.0–29.2)
		*Clostridium*	5.7 (2.5–7.2)	6.2 (3.9–8.3)	8.3 (3.1–11.8)
		*Eubacterium*	5.0 (1.4–7.1)	3.7 (3.2–9.6)	4.4 (3.7–8.9)
		*Ruminococcus*	0.7^A^ (0.5–6.0)	4.3^AB^ (2.3–5.0)	6.2^B^ (0.9–7.7)
		*Phascolarctobacterium*	0.5 (0.0–1.6)	0.0 (0.0–0.7)	1.3 (0.0–2.8)
		*Parabacteroides*	2.0 (0.5–3.4)	1.6 (0.4–4.5)	1.8 (1.1–3.8)
		*Veillonella*	0.5 (0.0–2.5)	0.8 (0.0–3.2)	0.5 (0.0–1.3)
		*Butyrivibrio*	1.4 (0.2–2.4)	0.7 (0.3–1.6)	0.9 (0.3–1.7)
		*Akkermansia*	4.1 (0.0–7.7)	1.3 (0.2–13.4)	3.1 (1.0–7.7)
		*Bifidobacterium*	0.2 (0.0–4.0)	0.8 (0.2–2.4)	0.4 (0.0–1.2)
		*Lactobacillus*	0.0^A^ (0.0–0.3)	1.7^B^ (0.5–7.8)	0.8^BC^ (0.5–4.0)
		*Bacillus*	0.5 (0.0–1.8)	0.1 (0.0–0.2)	0.1 (0.0–0.5)
*Bifidobacterium breve* CNCM I-4035		*Bacteroides*	22.7 (6.9–29.8)	25.2 (14.2–42.2)	19.2 (7.1–25.2)
		*Faecalibacterium*	6.9 (5.2–10.3)	6.6 (4.4–11.3)	7.1 (4.1–8.1)
		*Alistipes*	5.5 (4.7–9.8)	5.5 (3.7–9.2)	4.9 (1.6–10.3)
		*Prevotella*	7.7 (0.0–19.8)	3.0 (0.1–18.4)	12.1 (6.4–41.2)
		*Clostridium*	10.5 (5.5–11.3)	7.2 (2.2–11.6)	6.3 (5.4–8.4)
		*Eubacterium*	4.8 (3.8–7.7)	3.7 (2.1–5.4)	4.2 (2.0–11.8)
		*Ruminococcus*	4.0 (0.6–8.7)	2.0 (0.2–5.6)	2.4 (1.5–3.4)
		*Phascolarctobacterium*	0.0 (0.0–0.0)	0.0 (0.0–0.0)	0.0 (0.0–0.0)
		*Parabacteroides*	2.1 (1.2–3.6)	2.3 (0.8–3.2)	2.3 (0.9–3.6)
		*Veillonella*	1.5 (0.3–2.2)	1.6 (0.0–1.6)	2.1 (0.8–2.6)
		*Butyrivibrio*	1.5 (0.6–3.7)	1.3 (0.6–3.2)	2.1 (1.0–2.6)
		*Akkermansia*	0.0 (0.0–0.2)	0.0 (0.0–0.0)	2.9 (0.0–8.1)
		*Bifidobacterium*	0.1 (0.0–0.5)	1.1 (0.0–2.8)	0.0 (0.0–0.0)
		*Lactobacillus*	0.0 (0.0–0.7)	0.0 (0.0–0.0)	0.0 (0.0–0.2)
		*Bacillus*	1.5 (0.5–3.9)	2.4 (0.0–5.4)	0.3 (0.1–0.6)
	*Bifidobacterium breve* CNCM I-4035 and *Lactobacillus rhamnosus* CNCM I-4036	*Bacteroides*	16.5 (12.0–38.7)	23.2 (12.8–50.0)	18.4 (8.8–49.6)
		*Faecalibacterium*	7.3 (2.2–9.8)	10.3 (7.9–13.4)	6.5 (2.4–14.3)
		*Alistipes*	4.2 (3.8–7.9)	5.5 (2.8–7.3)	4.8 (1.0–11.7)
		*Prevotella*	7.6 (5.3–23.1)	7.8 (0.1–17.6)	10.6 (1.0–12.8)
		*Clostridium*	8.4 (2.3–11.1)	4.4 (2.3–8.2)	7.1 (4.8–8.4)
		*Eubacterium*	4.4 (3.0–10.1)	3.9 (2.1–6.2)	6.4 (3.8–10.7)
		*Ruminococcus*	4.9 (1.7–10.2)	2.7 (0.3–3.6)	4.1 (0.8–7.3)
		*Phascolarctobacterium*	0.0 (0.0–0.0)	0.0 (0.0–1.4)	0.1 (0.0–0.7)
		*Parabacteroides*	3.7 (1.3–4.0)	3.1 (1.9–5.9)	4.1 (2.6–8.9)
		*Veillonella*	0.4 (0.2–2.1)	0.9 (0.1–1.6)	0.5 (0.3–1.1)
		*Butyrivibrio*	1.3 (0.9–3.0)	1.3 (0.4–1.8)	1.3 (0.7–2.1)
		*Akkermansia*	0.8 (0.0–7.1)	0.1 (0.0–3.3)	0.0 (0.0–0.9)
		*Bifidobacterium*	0.4 (0.2–0.6)	0.3 (0.0–2.0)	0.4 (0.2–0.6)
		*Lactobacillus*	0.3 (0.0–0.5)	0.2 (0.0–0.7)	0.0 (0.0–1.6)
		*Bacillus*	0.5 (0.0–3.3)	0.5 (0.0–3.3)	0.0 (0.0–0.5)
	*Lactobacillus paracasei* CNCM I-4034	*Bacteroides*	23.6 (19.7–46.0)	21.3 (6.0–26.6)	21.8 (10.1–50.4)
		*Faecalibacterium*	9.6 (8.3–18.3)	9.8 (3.8–11.5)	10.3 (8.0–13.5)
		*Alistipes*	5.6 (3.5–12.8)	8.5 (2.2–11.5)	5.9 (3.8–8.9)
		*Prevotella*	0.1 (0.0–14.2)	2.4 (0.0–22.0)	0.0 (0.0–19.1)
		*Clostridium*	5.4 (3.1–7.4)	6.4 (5.7–8.3)	6.1 (4.0–7.5)
		*Eubacterium*	8.7 (3.8–13.8)	5.5 (3.6–8.3)	7.1 (5.6–7.8)
		*Ruminococcus*	2.4 (1.3–5.2)	2.7 (2.0–12.5)	3.4 (1.8–4.7)
		*Phascolarctobacterium*	1.0 (0.0–4.7)	0.6 (0.0–2.7)	0.1 (0.0–2.7)
		*Parabacteroides*	2.2^AC^ (0.0–3.4)	2.3^A^ (1.1–4.3)	1.3^BC^ (0.0–1.7)
		*Veillonella*	0.3 (0.0–3.1)	0.7 (0.0–1.5)	0.7 (0.0–1.4)
		*Butyrivibrio*	1.4 (0.7–3.3)	0.6 (0.6–2.7)	1.6 (1.2–1.9)
		*Akkermansia*	0.0 (0.0–7.0)	0.1 (0.0–0.1)	0.0 (0.0–0.1)
		*Bifidobacterium*	0.0 (0.0–1.7)	0.2 (0.0–1.9)	0.3 (0.0–1.1)
		*Lactobacillus*	0.0 (0.0–0.6)	0.1 (0.0–1.2)	0.2 (0.0–2.6)
		*Bacillus*	0.2 (0.0–0.8)	0.2 (0.0–2.1)	0.7 (0.0–3.1)

Values are median and range. Labeled medians without a common letter differ. *P* < 0.05. *t*_1_, first 15-day washout period; *t*_2_, intervention period; and *t*_3_, second washout for another 15 days.

The administration of *L. rhamnosus* CNCM I-4036 significantly increased the abundance of the *Lactobacillus* genus, which remained elevated after the second washout. Additionally, the *Ruminococcus* genus was significantly increased in the volunteers who received this strain after the second washout.

The *Parabacteroides* genus was significantly increased in the healthy volunteers who received *L. paracasei* CNCM I-4034 after the second washout. Furthermore, treatments with *L. paracasei* CNCM I-4034 or *L. rhamnosus* CNCM I-4036 significantly increased the Shannon index at the end of the intervention.

### 3.2. Impact of Lactobacillus rhamnosus CNCM I-4036 on the Colon Microbiota Compositions of Healthy Volunteers

**Figure 2:**
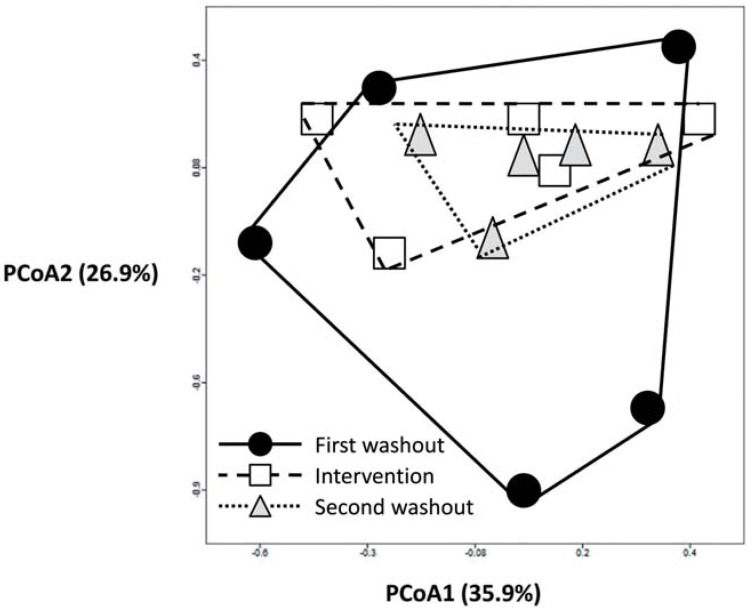
The impacts of *L. rhamnosus* CNCM I-4036 consumption on the intestinal microbiota. The first washout, intervention and second washout refer to the different *L. rhamnosus* CNCM I-4036 intake phases. Principal component analysis (PCoA) score plots of the different *L. rhamnosus* CNCM I-4036 intake phases are shown. The data were compared with those in the Ribosomal Database Project (RDP) using a maximum e-value of 10^−5^, a minimum identity of 75%, and a minimum alignment length of 15 measured in bp for RNA databases. The data were normalized to values between 0 and 1, and Euclidean distance was measured in the construction of PCoA plots. Each figure represents the composition of the intestinal microbiota of one healthy volunteer.

We next focused on *L. rhamnosus* CNCM I-4036 because it was the only probiotic strain that modified the colon microbiota (Table 2). To explore the manner by which the intestinal microbiota responds to *L. rhamnosus* CNCM I-4036 intake, we performed PCoA (Figure 2) to assess the fecal samples prior to the administration of the strain (first washout), after 30 days of its consumption (intervention) and after another washout (second washout). An apparent clustering pattern was identified for most of the subjects during the different *L. rhamnosus* CNCM I-4036 phases. A large amount of data points shifted from the external sides of the score plot before the *L. rhamnosus* CNCM I-4036 intake phase (first washout, black circles) to the center of the graph after the treatment was stopped (intervention, white squares) and at 15 days after the intervention (second washout, grey triangles), as shown in Figure 2. A PERMANOVA analysis showed statistically significant differences in *t*_1_ *vs. t*_2_, and *t*_1_ *vs. t*_3_ (*P* < 0.05).

**Figure 3:**
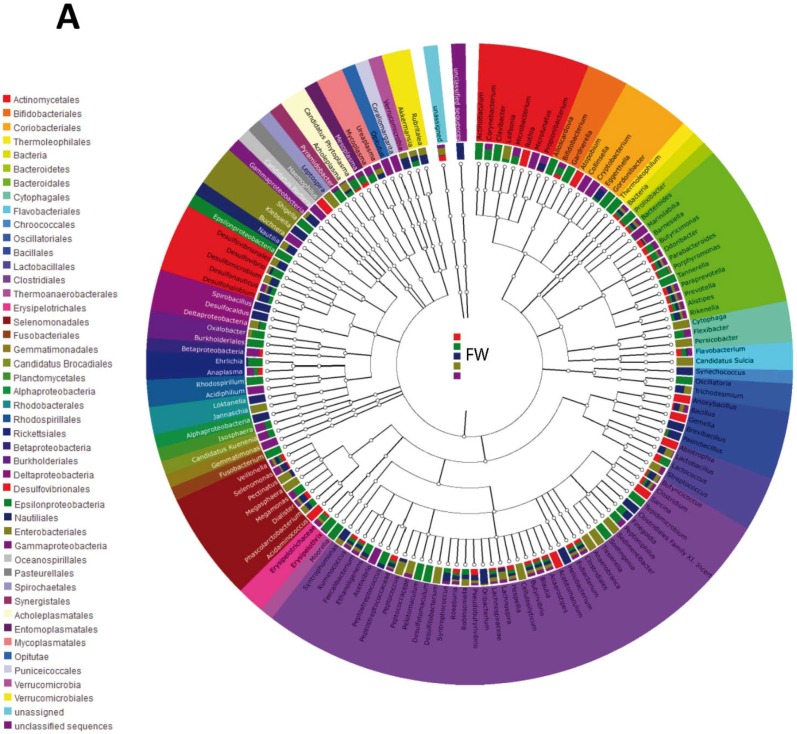

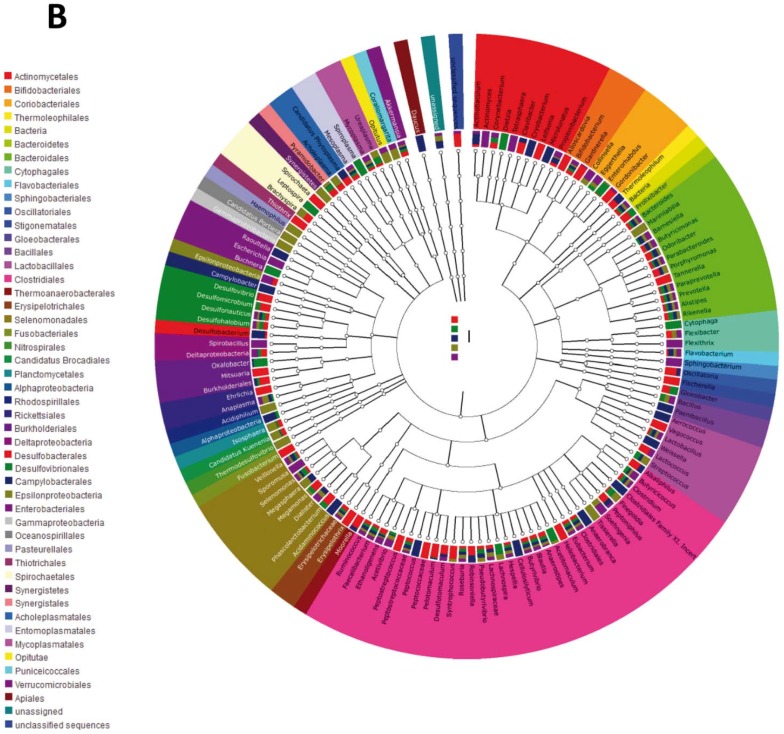

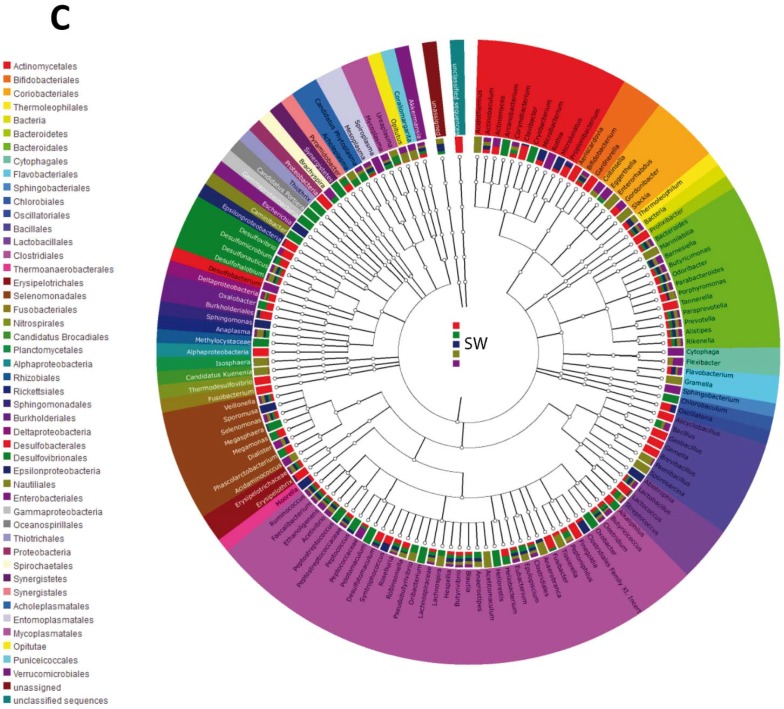
Tree diagram of the different *L. rhamnosus* CNCM I-4036 intake phases. (**A**) First washout. (**B**) Intervention. (**C**) Second washout. Each tree diagram represents the composition of the intestinal microbiota of five healthy volunteers who received *L. rhamnosus* CNCM I-4036. Colors represent orders. FW, First washout; I, intervention; and SW, second washout.

The tree diagram (Figure 3A–C) revealed differences in the three time points. The first washout (Figure 3A) presented a characteristic composition at the order level. The orders *Gemmatimonadales*, *Rhodobacterales*, and *Chroococcales* disappeared after the intervention and also after the second washout. The order *Nautilales* also vanished after the intervention but was detected again after the second washout (*t*_3_).

After 30 days of intervention with the strain *L. rhamnosus* CNCM I-4036, the *Sphingobacteriales*, *Nitrospirales*, *Desulfobacterales*, *Thiotrichales*, and *Synergistetes* orders were detected in the fecal samples (Figure 3B). All of these orders remained in the samples after the second washout.

Finally, the second washout (Figure 3C) presented five new orders (*Chlorobiales*, *Rhizobiales*, *Sphingomonadales*, *Proteobacteria*, and *Acholeplasmastales*) compared with the initial time point.

## 4. Discussion

In a recent multicenter, randomized, double-blind, placebo-controlled trial (SETOPROB), we have described that *L. paracasei* CNCM I-4034, *B. breve* CNCM I-4035 and *L. rhamnosus* CNCM I-4036 administration modifies the bacterial populations in fecal samples obtained from volunteers, as evidenced by real-time PCR and fluorescence *in situ* hybridization (FISH). Some of these changes were transient, whereas others were stable [15]. These two methodological evaluations only measured 10 bacterial groups; thus, their findings were not representative of the true colon microbiota composition following probiotic administration. Advancements in gene-sequencing technologies, as well as the increased availability of powerful bioinformatics tools, have enabled novel insights into the composition of the human colon microbiota and the effects of microbial communities on human physiology and disease. Studies using these technologies have indicated that dysbiosis (*i.e.*, an abnormal microbiota composition) and a decreased complexity of the gut microbial ecosystem are common features in patients with Crohn’s disease or ulcerative colitis [21]. The pyrosequencing analysis performed in the present study allowed for the determination of both the entire phylogenetic spectrum and the flexibility to analyze populations at different taxonomic levels.

Although some studies have suggested that bacterial taxa in the gut are continuously distributed, recent studies have identified three robust categories, termed enterotypes, on the basis of the abundances of key bacterial genera in adults [22,23,24]. These are the *Bacteroides*-, the Prevotella- and the Ruminococcus-dominated enterotypes, profiles which may, in part, be determined by long-term nutritional habits [20,24]. Our results with regard to the four major phyla (*Firmicutes*, *Bacteroidetes*, *Proteobacteria* and *Actinobacteria*) were similar to those obtained by Zhang *et al.* [25] and Ferrario *et al*. [8] in adult volunteers who received probiotics and to the findings of Azad *et al*. [26] in healthy infants.

*Bacteroides* was the most abundant genus in all of the study groups, but only those individuals who received *L. rhamnosus* CNCM I-4036 or *L. paracasei* CNCM I-4034 showed significant changes in their fecal compositions. Additionally, the Shannon indices were significantly increased at the end of the intervention in these two groups.

In the SETOPROB study, we found that healthy adults who received these two *Lactobacillus* strains showed decreases in pro-inflammatory cytokines together with increases in anti-inflammatory cytokines [15]. In addition, cell culture studies performed by Bermudez-Brito *et al*. [27,28] have shown that *L. paracasei* CNCM I-4034, *B. breve* CNCM I-4035 and *L. rhamnosus* CNCM I-4036 inhibit the production of pro-inflammatory cytokines and chemokines by human intestinal dendritic cells challenged with pathogenic bacteria, and that these effects seem to be mediated through the decreased expression of toll-like receptor (TLR)-1, TLR-5 and TLR-9 [27,28].

We considered the impact of *L. rhamnosus* CNCM I-4036 on the colon microbiota of healthy volunteers because previous data have suggested that the administration of this strain to volunteers for 30 days results in its intestinal colonization [15]. Our results revealed a significant increase in the *Lactobacillus* genus after the 30-day intervention with this strain, and its level remained elevated after the second washout. These important modifications in the colon microbiota detected with high-throughput 16S ribosomal RNA gene sequencing techniques were not observed in the previous FISH analysis [15].

Additionally, we discovered an apparent clustering pattern after the administration of *L. rhamnosus* CNCM I-4036. The data points shifted from the external sides of the score plot to the center of the graph, as shown in Figure 2. The initial colon microbiota compositions, represented by black circles, were altered in all of the healthy adults who received *L. rhamnosus* CNCM I-4036, becoming similar to one another after the intervention period (white squares). These alterations in the colon microbiota remained in the absence of probiotic administration (grey triangles) (Figure 2). We are aware of the limitations of the PERMANOVA analysis that we used to ascertain whether these changes were statistically significant: (1) the number of factors must not be greater than nine; (2) the design must have replication within cells (*i.e.*, *n* ≥ 2); (3) the sample size must be equal within each cell (*i.e.*, balanced designs only); and (4) all cells in the design must be filled: no cells (combinations of factors given) can be missing.

Furthermore, the tree diagram revealed differences at the three study time points at the order level. Some orders that were initially present in the colon microbiota disappeared after the administration of *L. rhamnosus* CNCM I-4036, whereas other orders, such as *Sphingobacteriales*, *Nitrospirales*, *Desulfobacterales*, *Thiotrichales*, and *Synergistetes*, were detected. These analyses confirm the impact of *L. rhamnosus* CNCM I-4036 on the colon microbiota. Similarly, Zhang *et al.* [25] found that the consumption of *Lactobacillus casei* significantly alters the composition of intestinal microbiota and colon microbiota diversity, and Ferrario *et al*. [8] have demonstrated that the intake of *L. paracasei* DG increases the *Blautia/Coprococcus* ratio, which can potentially confer a health benefit to the host [29,30].

Gender differences in microbial patterns induced by the intake of the probiotic strains cannot be excluded due to the small number of subjects. We are aware of the limitations of the small sample size of our study. However, although small, this size was similar to that described elsewhere [8,25,26]. Future trials enrolling a greater number of healthy adults would strengthen these preliminary results.

## 5. Conclusions

In summary, results obtained demonstrate that the intake of *Lactobacillus paracasei* CNCM I-4034, *Bifidobacterium breve* CNCM I-4035 and *Lactobacillus rhamnosus* CNCM I-4036 induced changes in the colon microbiota. Overall, our results warrant further studies to ascertain whether the modification of the microbiota composition might explain the immunomodulatory effects previously described for these probiotic strains.
